# Danger Zones of the Gluteal Anatomy: Improving the Safety Profile of the Gluteal Fat Grafting

**DOI:** 10.1007/s00266-023-03824-y

**Published:** 2024-02-01

**Authors:** Ana Cristina Seabra Robalo Gomes Jorge, You-Shan Feng, Adelana Santos Stahl, Gerd Grözinger, Konstantin Nikolaou, Matthias Glanemann, Adrien Daigeler, Stéphane Stahl

**Affiliations:** 1 Department of General, Visceral, Vascular, and Pediatric Surgery, Saarland University Hospital, Kirrberger Straße, 66421 Homburg, Saarland Germany; 2 Institute for Clinical Epidemiology and Applied Biometrics, Medical University of Tübingen, Tübingen, Germany; 3 CenterPlast private practice, Bahnhofstraße 36, 66111 Saarbrücken, Germany; 4 Department of Diagnostic and Interventional Radiology, University Hospital of Tübingen, Tübingen, Germany; 5 Department of Hand, Plastic, Reconstructive and Burn Surgery, BG Clinic Tübingen, Tübingen, Germany

**Keywords:** Gluteal fat grafting, Brazilian butt lift, Buttock augmentation, Gluteoplasty, Fat embolism, Fat transfer

## Abstract

**Introduction:**

Knowledge of the vascular anatomy is critical to performing safe gluteal surgery. To date, only the course of the main blood vessels within the muscles has been outlined. These findings are based on MRI and CTA images that do not conform to a topographically standardized and normalized probability distribution.

**Objectives:**

The aim of this study was to develop a three-dimensional mapping of the gluteal zones of high vascular density in relation to anatomical landmarks.

**Materials and Methods:**

This single-center retrospective cohort analysis comprised all consecutive patients who underwent cone-beam computed tomography (CBCT) scans between January 2016 and October 2021. The location of blood vessels in the gluteal region was allometrically normalized in relation to anatomical landmarks. Moreover, the caliber and area of the blood vessels were assessed.

**Results:**

CBCT scans of 32 patients with an average age of 64 ± 12 years (range 34–87 years) were included. Fifty-three percent were female. The median [IQR] caliber of the intramuscular gluteal vessels was 1.47 [1.15–1.88] mm, significantly greater than that of the subcutaneous vessels 1.09 [0.72–1.44] mm (*p *< 0.001). Vascular density was higher intramuscularly, as 4.5% of the area of the muscle was occupied by blood vessels, as opposed to 0.3% in the adipose tissue.

**Conclusion:**

The analysis of the CBCT scans showed a higher vascular density and larger vessels intramuscularly. We, therefore, recommend the injection of autologous fat merely to the subcutaneous plane.

**Level of Evidence II:**

This journal requires that authors assign a level of evidence to each article. For a full description of these Evidence-Based Medicine ratings, please refer to the Table of Contents or the online Instructions to Authors www.springer.com/00266.

## Most Important Information

- Our study pioneers the measurement of the diameter of all gluteal vessels and presents a three-dimensional allometrically standardized representation of the topographical gluteal anatomy.
- Intramuscular vessels had a significantly larger caliber than subcutaneous vessels (*p*  <  0.001).
- The largest arteries were located intramuscularly, either at the level of or just above the greater sciatic foramen or at the uppermost level of the pubic symphysis.
- Our results provide validation for the recommendations of the British Association of Aesthetic Plastic Surgeons (BAAPS), which advise limiting injections to the subcutaneous layer.

## Introduction

Knowledge of microvascular anatomy is crucial for conducting safe reconstructive surgery [1, 2]. The occurrence of life-threatening fat embolisms is still a matter of great concern [3, 4], despite efforts to deepen the knowledge of gluteal microvascular anatomy.

Two prior in vivo studies, using 40 CTA [2] and 8 MRI venographies [5], provided valuable information on the trajectory and diameter of the superior and inferior gluteal arteries (SGA and IGA), presenting a two-dimensional mapping of gluteal vessels. However, there remains a notable knowledge gap regarding the caliber and distribution of all gluteal vessels in an allometrically standardized three-dimensional representation. Moreover, their methodological approach is less robust than the one we propose, as these studies do not provide a comprehensive portrayal of the probabilities associated with various vascular courses. We also pioneer at measuring the diameter and recording the location of subcutaneous gluteal vessels.

In order to reduce the incidence of fatal and nonfatal pulmonary fat embolisms, the American Society of Plastic Surgeons (ASPS) [6, 7] and several authors recommend limiting the fat transfer to the subcutaneous plane [6, 8, 9], where fewer and smaller vessels are located [6, 8, 9]. However, a systematic review found it did not reduce the complication rate significantly in comparison to the injection into the intramuscular and subcutaneous planes (*p* = 0.059) [10].

This study aimed to evaluate gluteal vessel location and create a standardized three-dimensional, easily reproducible system for representing areas of greater vascular density. This system was designed in relation to palpable bone landmarks in order to guide plastic surgeons during the procedure.

## Material and Methods

### Study Sample

This retrospective study was approved by the Ethics Committee of the University of Tübingen (Project No. 276/2020BO2). Informed consent was waived.

We evaluated all consecutive pelvic CBCT examinations from January 2016 to October 2021, excluding patients with peripheral artery disease, scans with significant artifacts, unclear vascular delineation, or missing anatomical landmarks (specifically, the posterior superior iliac spine (PSIS)). We assessed patient age, gender, anthropometrics (height, weight, and BMI), CBCT indication, and secondary diagnoses for patients aged 18 or older.

### Image Acquisition of Cone-Beam CT scans

A robotic digital subtraction angiography system (Artis Zeego Q, VE 40 A, Siemens, Forchheim, Germany) was used for diagnostic angiography and endovascular procedures. A local anesthetic was applied and a 19-G needle was inserted into the common femoral artery, followed by a 4-Fr sheath (Terumo, Belgium). Next, 30 mL of 80% diluted contrast medium (Ultravist 370, Bayer Schering, Germany) was injected into a 4-Fr pigtail catheter in the infrarenal area for aortography (2 frames/s) at 15 mL/s rate.

Then, pelvis CBCT images were acquired with patients holding their breath and arms above their heads to reduce streak artifacts. A 50% diluted contrast medium (Ultravist 300) was administered at 8 mL/s for 10s, with a 6-second rotation time and a 60°/s detector speed. The settings were 90 kVp, 48 cm field of view, and a 512 × 512 voxel matrix.

CBCT data were transferred to a workstation (syngo XWP, Siemens Healthineers); afterward, CT-like axial images with a 0.5 mm voxel size were automatically reconstructed. Planes were predefined to have a 10mm thickness.

### Anatomic Boundaries and Image Assessment

**Fig. 1:**
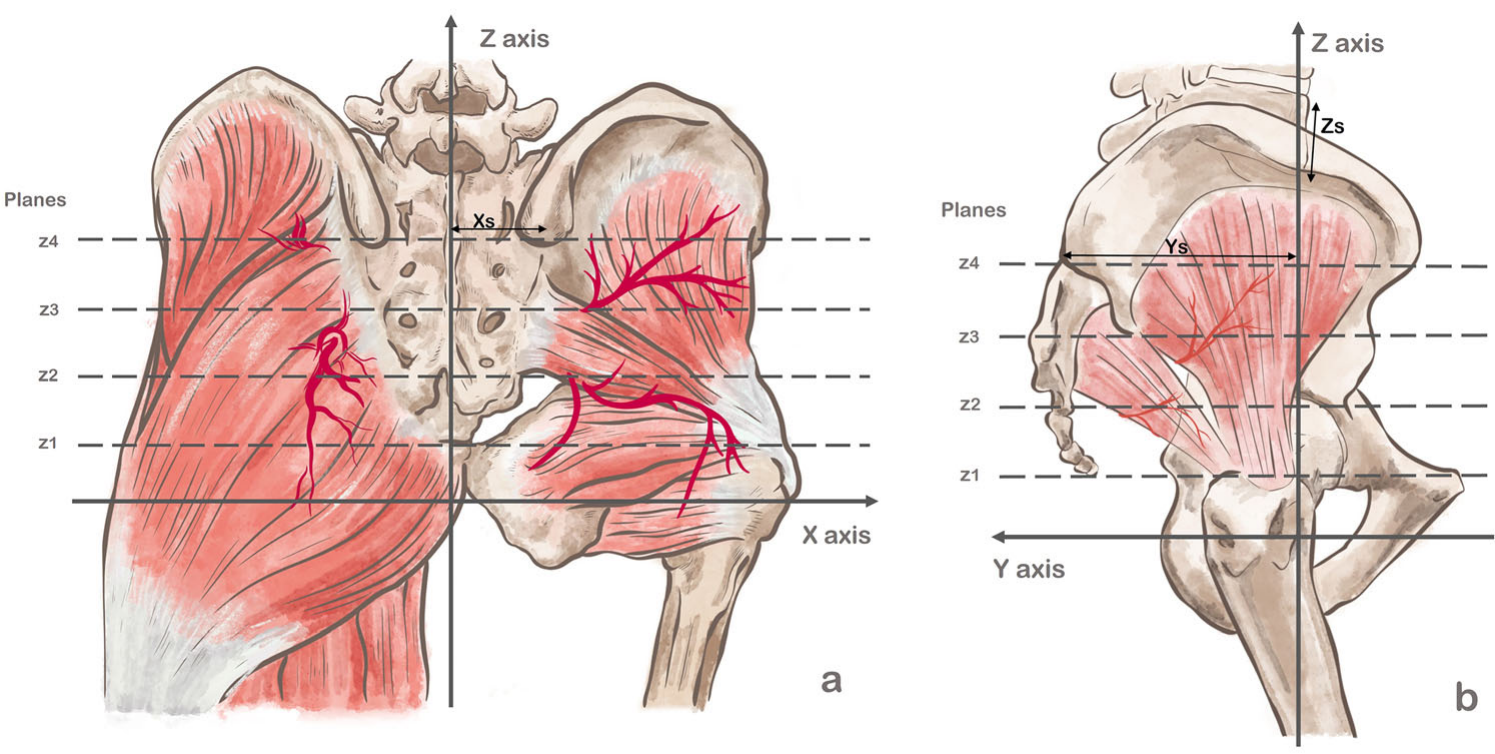
Representation of the four equidistant horizontal planes (*z*1, *z*2, *z*3, *z*4) and the 3 axes (x, y, z) (**a** and **b**). Xs represents the distance between the PSIS and the midline and Ys represents the distance between the midpoint between both PSIS and the lower anterior corner of the fifth lumbar vertebra

Lumbar spine anatomy is a reliable predictor of the human stature [11, 12], particularly the anterior height of the fifth lumbar vertebra [12]. To standardize the gluteal evaluation, we defined the region of interest’s height as the distance between PSIS and the upper pubic symphysis border, allocating four equidistant horizontal planes (*z*1*, z*2, *z*3, *z*4) (Fig. 1).

Our coordinate system employed a vertical axis (*z*) crossing the anterocaudal corner of the fifth vertebral body, an anteroposterior axis (*y*) intersecting the midpoint between PSIS (dividing planes into left and right), and a transverse axis (*x*) forming a 90° angle with the other axes and partitioning planes into anterior and posterior sections.

Standardization of the coordinates was based on predefined distances (Fig. 1):

- *X*_*s*_ denotes the distance between PSIS and the midline, accounting for pelvic dimorphism, as females have wider pelvises [13] and greater sacrum breadths than males (*p* = 0.008) [14].
- *Y*_*s*_ signifies the distance between the midline point between PSIS and the lower anterior corner of the fifth vertebral body, as lumbar vertebral anterior-posterior diameters are reliable predictors of stature [11, 15] and exhibit no sexual dimorphism (*p *> 0.05) [16].
- *Z*_*s*_ represents the anterior height of the fifth lumbar vertebra and was employed to estimate pelvic height when the symphysis was not depicted in CBCT scans, due to its correlation with human stature [12].

Negative *x* values indicate the left patient side and positive *x* values the right side. Additionally, the maximal thickness of muscle and fat layers were recorded in each plane.

ImageJ, a software by the National Institutes of Health and the Laboratory for Optical and Computational Instrumentation (University of Wisconsin), was used to measure vessel calibers.

### Statistical Analysis

We used mean and standard deviation for normally distributed quantitative data and median with interquartile range for asymmetric data. SPSS version 28 (SPSS Inc, Chicago, Illinois, USA) was used to conduct the statistical tests. We assumed that the depth of the vessels (represented as distance to the centre of the coordinate axes) is linear based on preliminary descriptive results. To account for repeated measures within individuals and clusters, mixed models were used to assess the vascular diameter (dependent variable) and right/left side, planes, locations (intramuscular versus subcutaneous), depth, and gender (Table 4). Random intercept models with fixed effects were estimated using the restricted maximum likelihood (REML) with scaled identity error covariance. Because of the skewed distribution of the vascular diameters, these measurements were log transformed. Significance was set at *P *≤ 0.05.

## Results

### Demographic Data

**Fig. 2:**
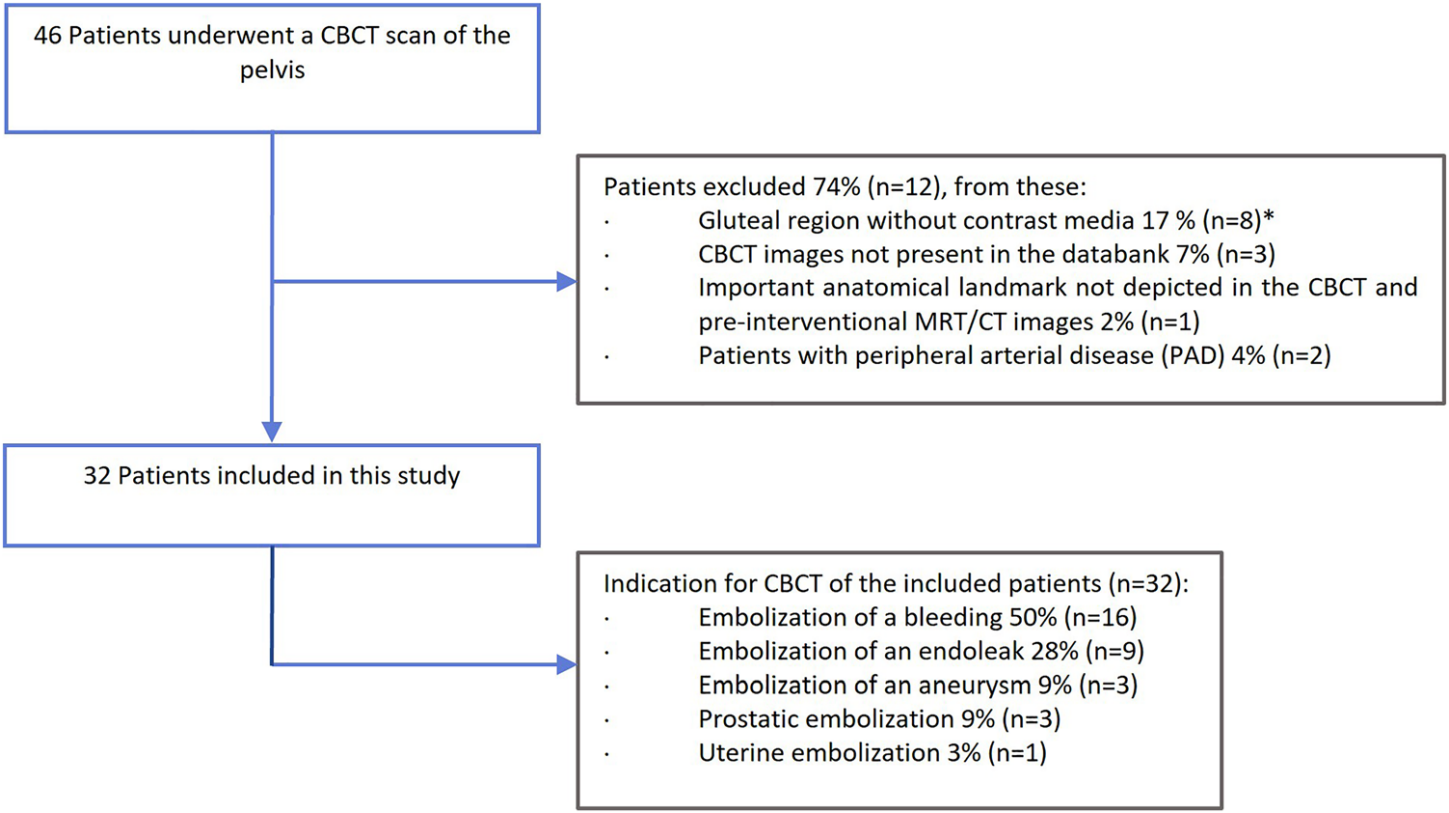
Diagram of the patient selection process. *One patient had two CBCT examinations, one with and one without contrast medium

Between January 2016 and October 2021, 46 patients underwent a CBCT examination of the pelvis performed at a single institution. Thirty-two of these patients met the inclusion criteria (Fig. 2). Approximately half of the patients was female and 91% of patients were 50 years of age or older (Table 1). Five patients (16%) were obese, and seven (22%) were overweight. The weight of two patients was unknown.

**Table 1:** Demographic data. *Embolisation of endoleaks were not included in this category, as the primary cause is a vascular malformation

	Patients (*n *= 32)	Female	Male
Number of patients	32 patients (100%)	17 patients (53%)	15 patients (47%)
Age	64 ± 12 (min 34, max 87)	62 ± 13 (min 34, max 81)	66 ± 11 (min 48, max 87)
BMI (kg/m^2^)	24.9 ± 4.6 (min 15.8, max 35.2) *	24.5 ± 4.6 (min 18.4, max 35.2)	25.3 ± 4.6 (min 15.8, max 33.1)
*Primary diagnoses*			
Carcinoma	13 (41%)	8 (47%)	5 (33%)
Vascular abnormality	15 (47%)	8 (47%)	7 (47%)
Coagulopathy	3 (9%)	2 (12%)	1 (7%)
Benign disease (e.g., BPH, Uterine myomas,…)	5 (16%)	2 (12%)	3 (20%)
Postoperative or postinterventional*	5 (16%)	1 (6%)	4 (27%)
Multimorbidity	23 (72%)	12 (71%)	11 (73%)

### Anatomical Data

Evaluation of the maximal thickness of the gluteal musculature and subcutaneous fatty tissue showed a moderate and weak positive and significant correlation with BMI, but neither with age nor with gender (Table 2).

**Table 2:** Pearson correlation between maximal thickness of muscle and adipose tissue and demographic factors. Student’s *t* test for comparison of the means of maximal thickness of muscle and fat in both genders

	Mean ± SD (mm)	BMI	Age	Gender
Maximal thickness of muscle	55.7 ± 18.3	*r* = 0.563 *p* < 0.001	*r* = −0.034 *p* = 0.631	*t*-statistic = −2.783 mean difference = −7.140 *p* = 0.368
Maximal thickness of subcutaneous fat tissue	42.5 ± 14.6	*r* = 0.279 *p* < 0.001	*r* = −0.074 *p* =0.318	*t*-statistic = 8.541 mean difference = 15.618 *p* = 0.657

The largest arteries were located intramuscularly at the level of or directly above the greater sciatic foramen (plane *z*_3_) and at the upper level of the pubic symphysis and femoral head (plane *z*_1_) and subcutaneously in the most caudal plane (plane *z*_1_) (Table 3, Fig. 1). The median (IQR) of all gluteal vessels was 1.43 [1.10–1.84] mm (Table 3).

**Table 3:** Median [IQR] number of perforators and caliber of the gluteal arteries according to location (mm)

According to plane	Number of arteries	Number of vascular segments (vascular trunk and branches)	Number of branches per artery	Caliber, mm		
				Intramuscular	Subcutaneous	Total
Plane 1	9 [7–14]	15.0 [11.0–20.8]	1.5 [1.2–1. 7]	1.54 [1.20–1.98]	1.15 [0.78–1.45]	1.48 [1.15–1.91]
Plane 2	8 [6–14]	13.0 [10.0–19.0]	1.4 [1.2–1.8]	1.41 [1.10–1.78]	1.06 [0.78–1.44]	1.39 [1.07–1.76]
Plane 3	9 [7–12]	15.0 [11.0–20.0]	1.5 [1.3–1.9]	1.55 [1.19–2.08]	1.00 [0.66–1.41]	1.51 [1.15–2.03]
Plane 4	8 [6–12]	10.0 [7.0–15.0]	1.3 [1.1–1.4]	1.35 [1.10 –1.67]	1.09 [0.70–1.45]	1.31 [1.06–1.64]
Total	9 [7–12]	13.0 [9.0–18.0]	1.4 [1.2–1.7]	1.47 [1.15–1.88]	1.09 [0.72–1.44]	1.43 [1.10–1.84]

This analysis showed a positive and significant correlation between the area of muscle and adipose tissue occupied by vessels (*p* < 0.001). The vascular density is higher intramuscularly, as 4.5% [3.0–6.4%] of the area of the muscle is occupied by blood vessels (*p* < 0.001), as opposed to 0.3% [0.0–1.1%] in the adipose tissue (*p* < 0.001).

Mixed model analysis of fixed effects revealed that plane, tissue type, and patient side are significant predictors of the vascular caliber (all *p*  <  0.001), while gender was not significant (*p *= 0.054). Likewise, depth of the vessel was a significant predictor of the caliber (*p* < 0.001). As represented in Tables 3 and 4, planes 1 and 3 had the largest vessels, while plane 2 is not significantly different from the reference group (plane 4). Intramuscular vessels had a significantly larger diameter than subcutaneous vessels (difference in median = 0.38 mm, *p* < 0.001). The gender-based disparity in vessel caliber was found to be statistically insignificant, with females exhibiting marginally thinner vessels compared to males (*p* = 0.054) (Table 4). Interestingly, the right buttock displayed larger vessels (*p* < 0.001) and a greater muscular area (*p *= 0.011) than the left side. Both sides exhibited outliers and a skewed distribution in vascular caliber.

**Table 4:** Mixed modeling of the vascular calibers in each subcategory (plane, side, gender, and tissue) in relation to the logarithm of the vascular caliber. To estimate the depth of the coordinate points distance to the center was used.

Parameter		Regression coefficient	*P* value	95% Confidence Interval
	Intercept	0.030	0.329	−0.031 to 0.091
Plane	Plane 1	0.080	< 0.001	0.057–0.103
	Plane 2	−0.005	0.653	–0.027 to 0.017
	Plane 3	0.130	< 0.001	0.110–0.150
	Plane 4	Reference		
Tissue type	Muscle tissue	0.338	< 0.001	0.314–0.362
	Subcutaneous fat tissue	Reference		
Gender	Female	−0.067	0.054	−0.134 to0.001
	Male	Reference		
Patient side	Right	0.047	< 0.001	0.033–0.062
	Left	Reference		
Depth of the vessel		−0.030	< 0.001	−0.045 to −0.014

## Discussion

The present study pioneers the measurement of the diameter of all gluteal vessels and presents a three-dimensional allometrically standardized representation of the topographical anatomy of the gluteal vessels in relation to bone landmarks. This map is tailored for applications in plastic surgery.

Our findings reveal a scarcity of subcutaneous blood vessels (Fig. 2) and smaller vessel sizes (Table 3). While a systematic review indicated no significant decrease in complication rates with subcutaneous-only injections compared to intramuscular and subcutaneous (*p *= 0.059) [10], fat embolism was reported in only one article [10]. To date, only survey results have been published [17], which carry an inherent bias.

**Fig. 3:**
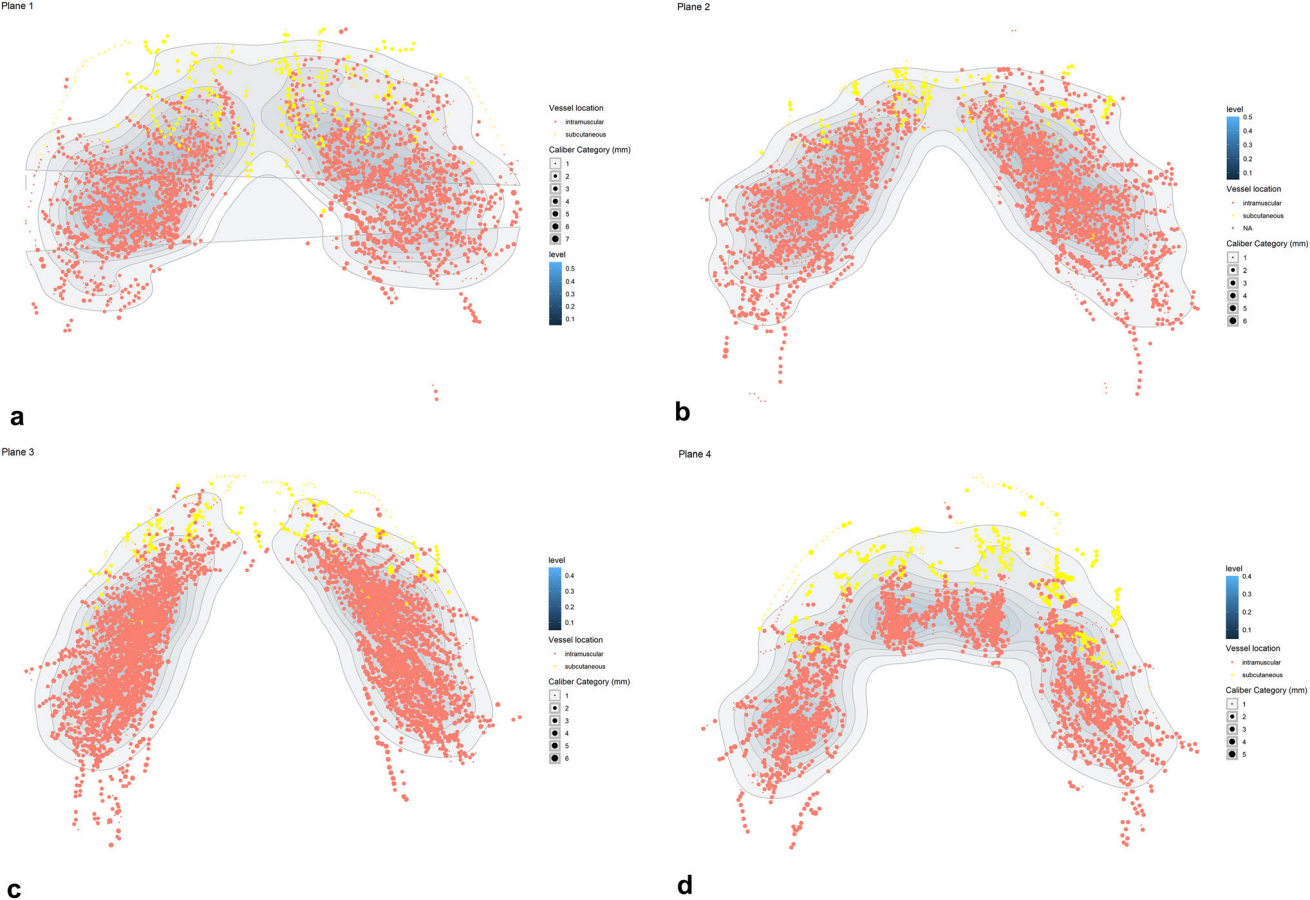
Vascular distribution according to plane. The level represents the density of points (decimal representation)

Several studies explored the gluteal anatomy to improve the safety of gluteal augmentation. A “danger triangle,” amid PSIS, greater trochanter, and ischial tuberosity, housing major gluteal vessels and the sciatic nerve has been described in the literature [8]. However, some assert that the subcutaneous plane is the only safe area for fat grafting [7, 18]. Our results demonstrate an even distribution of the intramuscular vessels and scarcer subcutaneous vessels. Lateral subcutaneous vessels were not visible due to the limited CBCT scan field of view (Fig. 3).

Accidental injection of fat into or near injured veins is a well-documented mechanism of fat embolism [8, 19–21]. CBCT scans depicted gluteal arterial anatomy. However, vessels typically travel together [22] and, in normovolemic patients, the diameter of the femoral vein and artery are similar [23]. Level 5 evidence advises intraoperative ultrasound for subcutaneous fat injection to reduce embolism risk [7, 19, 24], as it lacks large veins. Vein size may predict pulmonary fat embolism risk more than injected fat amount [25, 26]. Further research is required to correlate vein cannulation likelihood with higher intramuscular vascular density.

The gluteal region displays a tissue-dependent vascularity. Unlike gluteal augmentation, breast lipofilling is overwhelmingly done in the subcutaneous plane. A similar vascular distribution in the breast would explain why breast lipofilling lacks analogous risks and should discourage from lipofilling into the pectoralis muscle.

Only two other in vivo studies investigated the gluteal vascular anatomy. Vigato et al. [2] reported mean calibers of the largest SGA and IGA perforators (1.3 ± 0.4 mm and 1.2 ± 0.4 mm, correspondingly), aligning with our findings (gluteal vessels' median caliber: 1.43 [1.10–1.84]). Vigato et al. [2] plotted vessel data using the lumbosacral joint to greater trochanter distance as reference, without standardizing the height of the pelvis according to body size or the depth of the vessels in relation to the skin surface. Body mass index has a positive and statistically significant correlation with muscle and subcutaneous fat layer thickness (Table 2). Lower BMI patients might face heightened risks from unintended deep injections if the stated correlation is not taken into consideration.

Our results present medians and interquartile ranges due to skewed data, which may explain why the average diameter of the larger perforators is similar to the median caliber of all gluteal vessels. While the mentioned study investigated the location and number of other gluteal vessels (perforators of lumbar, lateral circumflex, internal pudendal, and profunda femoris arteries), their diameter within the intramuscular and subcutaneous course was not assessed. In our study, both tissue type and vascular depth proved to be reliable predictors of vascular diameter. However, we argue that tissue type supersedes the influence of depth, given that depth is contingent upon tissue type, while the inverse relationship does not hold true. Moreover, our adjusted model revealed that the disparities in vascular diameters across both tissue types were more pronounced than those associated with varying depths.

Turin et al. [5] reported SGV tributary diameters ranging from 1.5 to 2.5 mm on MRI venography, with subsequent branching into smaller veins (< 2 mm) within the muscle. In contrast, our research revealed intramuscular perforators often exceeding 2 mm in diameter. Notably, the aforementioned study did not specify the measurement positions, although vein diameter can decrease by up to 27% in the jackknife and 15% in lateral decubitus positions [5]. Adipose tissue’s incompressibility [27] implies that position changes are unlikely to affect subcutaneous vessel locations.

Both studies lack depth information relative to skin, limiting their practical value for surgeons. Areas with higher vascular density were the superior lateral region of the gluteus maximus muscle (SGA perforators) and the horizontal level of the greater trochanter and ischiadic tuberosity (IGA perforators) [2]. Our findings furnish empirical support for the guidelines of the British Association of Aesthetic Plastic Surgeons (BAAPS), which recommend limiting injections to the subcutaneous plane.

Females, older individuals, and those with smaller stature exhibit lower skeletal muscle mass [28]. Conversely, overweight elderly individuals exhibit resistance to muscle anabolic processes [29]. We found a positive correlation between muscle thickness and BMI (*r *= 0.563, *p* < 0.001) (Table 2). However, BMI's correlation with fat volume has limited significance, as it does not consider body composition, age, and gender. The intramuscular vessel area increases with gluteal muscle thickness (rho = 0.537, *p* < 0.001).

The calibers of lower limb arteries, with the exception of the arteria dorsalis pedis, did not exhibit any gender-specific differences [30]. Our study extends this finding to include gluteal arteries. Furthermore, the observed 5.4% difference in mean vascular diameter between sides aligns closely with the range of normal anatomical variations reported, which falls between 6 and 8% [30]. This variation may be attributed to the typical anatomical dominance of one limb side.

To our knowledge, this is the first plastic surgery study based on allometric normalization. We refrained from identifying the source artery, as its relevance to surgeons is negligible. CBCT demonstrated superior image quality than 3D-CTA [31, 32], high spatial resolution [32–35], strong blood vessel contrast [32], and lower radiation than MDCT [33]. CBCT is used for patients with inconclusive results on pre-interventional CTA and selective angiography to identify vessels for targeted embolization [32, 36]. The vessel-tracking software has a 92% sensitivity in detecting prostatic arteries [36, 37], surpassing selective angiography and/or CTA in diagnostic confidence [32].

The retention of fat graft volume is influenced by physiological, anatomical, technical, and patient-specific factors. Regarding physiological aspects, graft survival depends on the diffusion of nutrients and oxygen [38]. Consequently, it is reasonable to infer that the success of a fat graft is higher in well-vascularized tissues, such as muscle, in comparison to adipose tissue. However, there is currently no supporting evidence suggesting that the success of a fat graft is equivalent to that of a skin graft, indicating that grafting into muscle is not necessarily superior to grafting into adipose tissue. A study on subcutaneous fat transfer outcomes demonstrated a 12-month postoperative absorption rate of 18.2%, aligning with previous studies on intramuscular fat injection, which showed absorption rates ranging between 20 and 36% [4]. Regarding anatomical factors, fat graft requires a uniform distribution to prevent the formation of fat cysts. Therefore, a larger recipient volume allows for the transplantation of a greater amount of fat graft. Larger volume gains, when fat is injected into the muscle may simply be attributed to the fact that the recipient volume of muscle and subcutaneous fat is greater that subcutaneous fat alone. Furthermore, the graft survival and the subsequent volume gain are notably influenced by factors such as patients' age [39] and smoking habits [40]. Finally, the tumescent solution [41], the time between harvesting and infiltration [42], the isolation of fat cells and stem cells from the lipoaspirate [43] as well as the negative pressure during aspiration influence graft survival and volume gain [44]. Based on current evidence, the risks of fat graft to the gluteus muscle outweigh the benefits. Since the recipient’s volume increases after every successful fat graft, the achievable volume gain increases from one procedure to another. Safe large volume augmentations can be achieved by optimizing every factor that influences graft survival, with the option to repeat the procedure at the patient's discretion.

Study limitations include an older study population than that of gluteal augmentation [4, 10, 45, 46], due to ethical concerns that apply to all vascular research studies. Although the male percentage was higher than in the reported 1.8% male patients [10], gender-based vascular caliber differences were statistically insignificant (*p *= 0.054).

The CBCT scans were performed in supine position. Even though the IGV and SGV calibers diminish in the gluteal fat grafting positions (“jackknife” [8], prone [4, 10], lateral decubitus [47]) when compared to the prone position [5]. We reason that arteries are unsusceptible to the diameter variation that occurs with different positions, because they are not influenced by blood pooling. To date, there has been no evidence of the existence of non-concomitant veins of the gluteal region.

## Conclusion

This article explores the gluteal arterial anatomy in relation to anatomical landmarks. Our findings show larger, more numerous intramuscular vessels than subcutaneous ones. We, therefore, support the recommendation of injecting autologous fat merely to the subcutaneous plane. Additionally, vessel-occupied tissue area correlates positively with the buttock area. Our results help improve the knowledge of gluteal microvascular anatomy, reducing fat embolism rates.
